# The role of attention in perceptual biases in patients with schizophrenia spectrum disorder and healthy controls

**DOI:** 10.1007/s00406-026-02317-8

**Published:** 2026-07-04

**Authors:** Alexander C. Schütz, Ogün Bolat, Kin Kori, Tilo Kircher, Benjamin Straube

**Affiliations:** 1 AG Sensomotorisches Lernen, Fachbereich Psychologie, Philipps-Universität Marburg, Marburg, Germany; 2 Center for Mind, Brain and Behavior, Philipps-Universität Marburg, Justus-Liebig-Universität Gießen, Technische Universität Darmstadt, Marburg, Germany; 3 Klinik für Psychiatrie und Psychotherapie, Philipps-Universität Marburg, Marburg, Germany

**Keywords:** Multistable perception, Schizophrenia, Priors, Individual differences, Motion perception

## Abstract

Ambiguous or uncertain sensory information is often resolved by incorporating prior assumptions about the most likely interpretation. Similar neural structures and a common visual environment typically lead to homogeneous priors in the population. Ambiguous motion displays are a rare exception as sustained idiosyncratic directional preferences have been observed. Patients with schizophrenia spectrum disorder (SSD) have been shown to rely less on perceptual priors in some contexts, yet their idiosyncratic directional preferences in ambiguous motion perception remain unexplored. Directional preferences were measured in two ambiguous displays: apparent motion and transparency-from-motion. To assess the strength and temporal stability of these preferences, we conducted measurements at two time points: first, during the acute phase of SSD in 32 hospitalized patients, and second, after symptom remission. A matched group of 18 healthy controls was also tested at comparable intervals. We found that patients exhibit idiosyncratic directional preferences that are similar in strength and stability as in healthy observers. Across patients and controls, the preference strength was positively correlated with performance in the d2-test, which measures selective and sustained attention. Apart from that, there was no evidence that the severity of positive or negative symptoms across time or the performance in a visuo-spatial working memory task would be related to the directional preferences. These findings suggest that certain perceptual priors can be preserved in SSD, regardless of symptom status, with attentional resources playing a crucial role in determining the strength and stability of directional preferences.

**Supplementary Information:**

The online version contains supplementary material available at https://doi.org/10.1007/s00406-026-02317-8.

## Introduction

The nature of our environment and our sensory organs can lead to uncertain and ambiguous sensory information. For instance, direct information about depth is lost in the 2D projection of the image on the retina. The brain has to infer the distal causes of the sensory input and it is believed that prior assumptions about the probability of different interpretations are used to overcome uncertainty and ambiguity [1–3]. Examples for such priors are the hollow-face-illusion, a case of binocular depth inversion [4, 5], in which a hollow-mask is consistently perceived as convex, or the light-from-above prior [6, 7], describing that object shape in 2D images is perceived such that it is compatible with light coming from the top. Often, the perception of such ambiguous stimuli is similar across observers, indicating that these priors are shared amongst the general population. Nevertheless, such priors can be modified by experience [8, 9] and can change over the course of development [10, 11].

Although there are many examples for homogeneous perception of ambiguous stimuli and therefore shared perceptual priors across the population, there are at least two striking exceptions with pronounced differences in the perceptual interpretation of ambiguous stimuli, which challenge traditional accounts of perceptual priors: first, perception of ambiguous stimuli in patients with mental disorders and second, perceptual preferences in ambiguous motion displays.

An altered development of internal models and use of priors has been hypothesized in patients with schizophrenia [for reviews see 12–14]. For instance, patients are less susceptible to some perceptual illusions [for reviews see 15–17]. Amongst them is binocular depth inversion [e.g. 18, 19], where patients are more likely to perceive a face mask as hollow than healthy observers. Also, patients are less likely to report the expected stimulus when the physical stimulation is weak or absent [20]. These findings suggest that under some conditions, perception in patients with schizophrenia is less influenced by prior information and more strongly driven by current sensory information than in healthy observers.

Exceeding the quantitative differences between patients and healthy observers, there are ambiguous motion displays that result in qualitative differences between observers. Examples are apparent motion (AM), where the visual stimulation is compatible with two opposite motion vectors [21, 22] and transparency-from-motion (TfM) [23–26, 22], where the depth ordering of two dot clouds moving in opposite direction is ambiguous. Observers exhibit preferences for a certain motion direction, which on the one hand vary substantially between observers, but on the other hand are stable over time within an individual [25, 21, 24, 27]. Notably, these directional preferences are independent between different ambiguous motion displays and between different effectors [22, 21]. Conceptually, these preferences can be framed as early sensory biases or as perceptual memory. While it is difficult to pinpoint them to a single origin, at least some of them arise quite early in visual processing [23].

In this study, we investigated if patients with schizophrenia spectrum disorder (SSD) show alterations in the directional preferences in ambiguous motion displays. If these directional preferences reflect perceptual priors and patients rely less on priors, patients should exhibit weaker or more volatile preferences compared to healthy controls. To test whether possible effects are state (current psychopathology) or trait (diagnosis per se) related, we tested the same patients twice, during acute symptoms and after remission, an approach that was rarely employed in previous studies.

## Methods

### Design

**Fig. 1:**
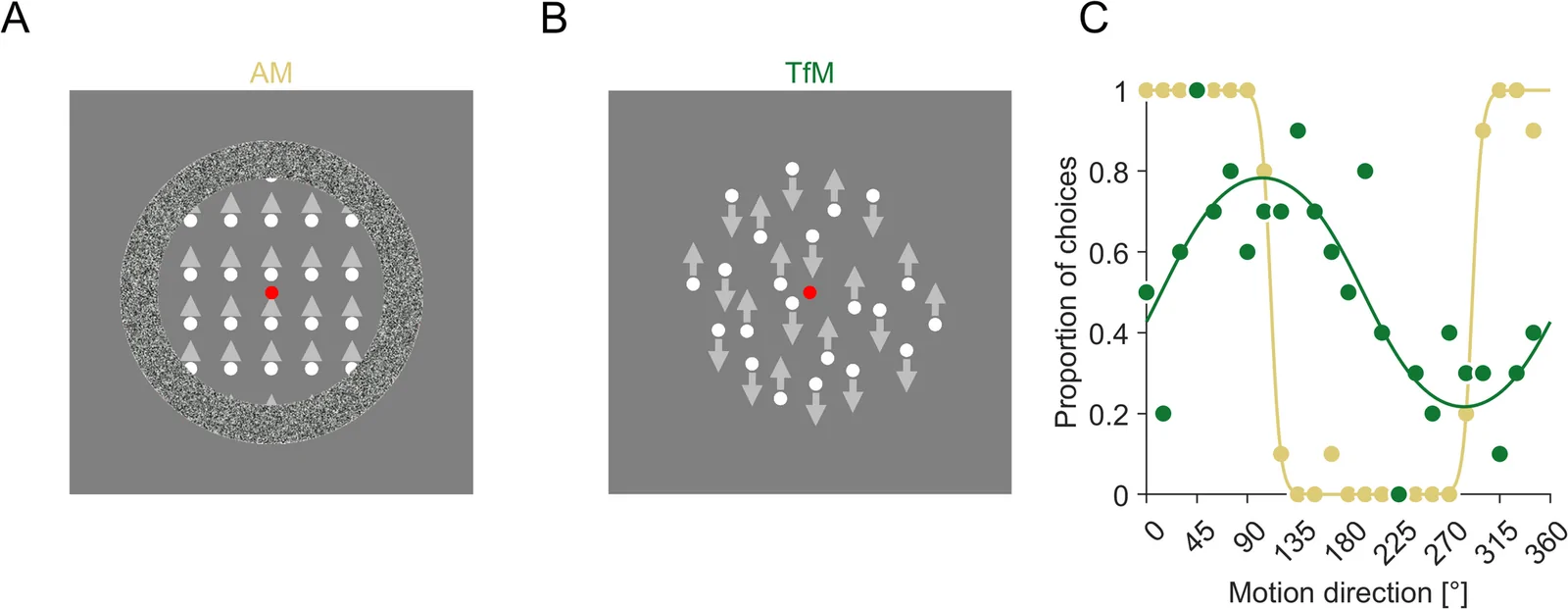
Stimuli and data analysis. **A**, Apparent motion (AM) task. Dots were arranged in a grid and step by half of the distance every 100 ms to generate ambiguous motion. **B**, Transparency-from-motion (TfM) task. Half of the dots moved in one direction and the other half in the opposite direction, resulting in the perception of two overlaid, transparent surfaces. A & B, Stimuli are not drawn to scale. Arrows indicate motion direction of dots. **C**, Perceptual responses of one participant in the first session of the AM (yellow) and the TfM task (green). The x-axis shows the motion axis of the ambiguous display. The y-axis shows the proportion a certain motion direction was reported. The line shows the model fit

We measured directional preferences in two ambiguous motion displays, AM and TfM, which have been used in previous studies on directional preferences [24, 23, 25, 22, 21]. In AM, a grid of dots is stepped by half of the grid distance (Fig. 1A), such that the correspondence between the two images is ambiguous [28]. In TfM, two overlapping dot clouds move in opposite directions (Fig. 1B), resulting in the illusory perception of depth, where one dot cloud is located behind the other [29, 30]. Directional preferences were measured in two sessions in patients, first when they were hospitalized due to a worsening of symptoms (acute phase) and a second time either three to four months later or when discharged from the hospital (partial/remission). A group of healthy control participants with a similar distribution of age, sex and education as the patients was also measured twice with comparable intervals (Table 1).

### Participants

We collected data from 38 patients with SSD and 18 healthy control participants. Patients were recruited at the Department of Psychiatry and Psychotherapy of Marburg University. Control participants were recruited through mailings at Marburg University and announcements at public places. Exclusion criteria for all participants were acute or chronic neurological or general medical illness and past or present drug or alcohol addiction. Additional exclusion criteria for healthy controls were history of psychiatric illness, personally or in first-degree relatives. We had to exclude data from one patient, who could not reliably discriminate the unambiguous control task (see below) and from five patients, who did not participate in the second session. Table 1 shows the characteristics of the final sample. The clinical diagnosis for patients was performed according to the structured clinical interview for DSM IV [German version, 31]. Symptoms were quantified with the scale of negative symptoms (SANS) [32] and the scale of positive symptoms (SAPS) [33], which are psychopathological rating scales to measure symptoms in schizophrenia. Twenty-one patients were diagnosed with schizophrenia (International Classification of Diseases, Tenth Revision [ICD-10] GM F20), two patients with acute polymorphic psychotic disorder (ICD-10 GM F23.0) and nine patients with schizoaffective disorder (ICD-10 GM F25.0). The study was conducted according to the principles of the Declaration of Helsinki (1964) and was approved by the ethics committee of the Department of Medicine at the University of Marburg (Study 22/19).

**Table 1:** Characteristics of the sample. Scale of positive symptoms (SAPS)

	Patients (*n* = 32)		Controls (*n* = 18)	
	1st session	2nd session	1st session	2nd session
Age (years)	39.03 ± 12.81 (19–61)		34.72 ± 11.75 (18–58)	
Sex ratio	11 female, 21 male		6 female, 12 male	
Days between sessions	53.38 ± 23.91 (21–106)		70.78 ± 35.72 (28–132)	
SAPS	32.69 ± 18.32 (5–76)	15.75 ± 11.97 (1–47)	–	–
SANS	30.59 ± 16.00 (7–64)	21.91 ± 14.06 (2–54)	–	–
CBTT	14.91 ± 3.39 (8–23)	17.31 ± 3.49 (8–25)	20.44 ± 2.48 (16–24)	20.89 ± 2.35 (17–25)
d2-test	109.19 ± 56.50 (9–241)	138.47 ± 53.80 (23–272)	166.22 ± 29.03 (129–251)	190.78 ± 30.07 (154–286)
Chlorpromazine equivalents (mg/d)	605.23 ± 476.32 (0–2023)	612.76 ± 405.23 (0–1380)^a^	–	–

Values denote averages ± standard deviations (minima – maxima).

Scale of negative symptoms (SANS), Corsi-Black-Tapping Task (CBTT)

^a^ indicates missing data for four patients

There were twice as many males than females in both groups, but this should not affect results as only very small gender effects for these ambiguous motion stimuli were reported in a large sample of 1000 participants [22]. The inter-session interval was slightly longer for healthy controls than for patients (t(48) = 2.061, *p* = 0.045, BF_10_ = 1.580). For patients, SANS (t(31) = 8.02, *p* < 0.0001, BF_10_ = 2.63*10^6^) and SAPS (t(31) = 6.76, *p* < 0.001, BF_10_ = 107262.62) values were significantly lower in the second than in the first session, indicating that patients’ symptoms subsided. For the CBTT, there was a significant interaction between group and session (F(1,48) = 10.632, *p* = 0.002): performance increased from the first to the second session for patients (t(31) = 6.28, *p* < 0.001, BF_10_ = 30165.19), but not for controls (t(17) = 1.054, *p* = 0.307, BF_10_ = 0.394, anecdotal evidence for the null hypothesis). For the d2-test, there were significant main effects of group (F(1,48) = 62.345, *p* < 0.001) and session (F(1,48) = 16.085, *p* < 0.001), but their interaction was not significant (F(1,48) = 0.480, *p* = 0.492). Hence, performance in the d2-test was lower in patients than in controls but similarly improved between session. 31 and 27 patients were on antipsychotic medication in the first and second session, respectively. Medication data are missing for four patients in the second session. The chlorpromazine equivalents [34] were not significantly different in the two sessions (t(27) = 0.603, *p* = 0.551, BF_10_ = 0.237).

### Apparatus and stimuli

As proxy measures of cognitive functioning, sustained attention was measured with the d2-test [35], which is a paper-pencil cancellation task and spatial visual working memory (VWM) was measured with the Corsi Block-Tapping Task (CBTT; 36), in which a visuo-spatial pattern has to be repeated.

The perceptual experiments were conducted on a Lenovo T480 laptop with a 14” display with a size of 30.9 × 17.4 cm (29.5 × 16.6 degrees of visual angle, dva), a resolution of 1920 × 1080 pixels and a refresh rate of 60 Hz. All. As the viewing distance was variable because it was not controlled by a head rest, all stimulus properties are specified in pixels and in dva at a typical viewing distance of 60 cm. The display was Gamma corrected to achieve a linear luminance output. The resulting luminance of black, gray and white pixels was 1.2, 134.8, and 281.1 cd/m², respectively. The experiments were controlled in Matlab 2018a (Mathworks Inc., Natick) via Psychtoolbox [37]. A combination of a cross and a bull’s eye in red color was used as fixation target [38].

### Apparent motion (AM)

For AM, dots with a size of 9 pixels (0.14 dva) were displayed in a square grid with a spacing of 52 pixels (0.8 dva). All dots were either black or white, randomized across trials. The dots were viewed through a circular aperture with a radius of 520 pixels (8 dva) at the screen center. The aperture had a noise pattern as border with a width of 130 pixels (2 dva) to facilitate the appearance of an aperture and the perception of AM [39, 21]. In the experimental block, the dots stepped half of the dot-distance every 100 ms, leading to an ambiguous motion of 260 pixels/s (4 dva/s) in the two possible directions. Directions from 0° to 165° in steps of 15° were tested 10 times. Participants had to judge in which direction the dots moved. In the control block, the dots stepped every frame by 4.3 pixels, leading to an unambiguous motion of 260 pixels/s (4 dva/s). Directions from 0° to 330° in steps of 30° were tested once.

### Transparency-from-motion (TfM)

For TfM, 178 black dots with a size of 13 pixels (0.2 dva) were displayed in a circular aperture with a radius of 488 pixels (7.5 dva) at the screen center. In the experimental block, half of the dots moved every frame in one direction by 5.4 pixels, leading to a speed of 325 pixels/s (5 dva/s). The remaining half of the dots moved in the opposite direction. Directions from 0° to 165° in steps of 15° were tested 10 times. Participants had to judge which motion direction they perceived in front, i.e., closer to them. In the control block, all dots moved in the same direction. Directions from 0° to 330° in steps of 30° were tested once.

### Procedure

Participants first completed the SKID interview, the d2 and the CBTT. Afterwards they performed the two perceptual tasks. In each of the two tasks, the experimental block preceded the control block. Every trial started with the presentation of the fixation target. After pressing the space bar, the fixation target remained on screen and the perceptual stimuli appeared for 600 ms. Afterwards, a response arrow was shown randomly in one of the two possible response directions. Participant could toggle the arrow’s direction with the plus key to match it to their perceived motion direction and the return key to confirm their selection and to proceed to the next trial. At the beginning of the experiment block, 12 demonstration trials with a longer motion duration of 1.2 s were shown. These were used to explain the task and to familiarize participants with the interface and were not included in the data analysis.

### Modelling and data analysis

In the perceptual tasks, we calculated how often each stimulus direction was selected (Fig. 1C). A cosine model was used to extract the preferred direction ($\:{\theta\:}_{m}$) and the preference strength (* b*):

1 $$\:y={10}^{b}\mathrm{cos}\left(\theta\:-{\theta\:}_{m}\right)$$

The preference strength was computed as an exponential parameter because values were more normally distributed.

The unconstrained model responses (* y*) were transformed to the scale of proportions between zero and one by a logit transformation:

2 $$\:p=\frac{{e}^{y}}{\left(1+{e}^{y}\right)}$$

Given our resolution of motion direction of 15°, we limited the value of *b* to a maximum of two. Higher values would lead to even steeper transitions between two data points that are not constrained by the data.

Reaction times were analysed only for trials in which the initial direction of the response arrow corresponded to the final selected direction. Reaction times below 200 ms and above 5000 ms were excluded for that analysis.

Data were analyzed with Bayesian ANCOVA with task, group and time as fixed factors, participant as random factor and d2 and CBTT as covariates. Only for patients, chlorpromazine equivalents as well as SANS and SAPS including their subscores were additional covariates. For the ANCOVA, all covariates were z-transformed. Uniform (ANCOVA) and default Cauchy priors (t-tests) were used. Circular correlation between the preferred directions were calculated with the Circstat toolbox [40]. The data analysis was conducted with Matlab 2022a (Mathworks, Inc., Natick) and JASP 0.18.1 [41].

## Results

We measured directional preferences in two ambiguous motion displays in patients with SSD and healthy control participants. Patients were measured twice, first during an acute phase at a stay in the hospital and a second time after remission. Control participants were measured twice at matching intervals (Table 1).

### Reaction times

**Fig. 2:**
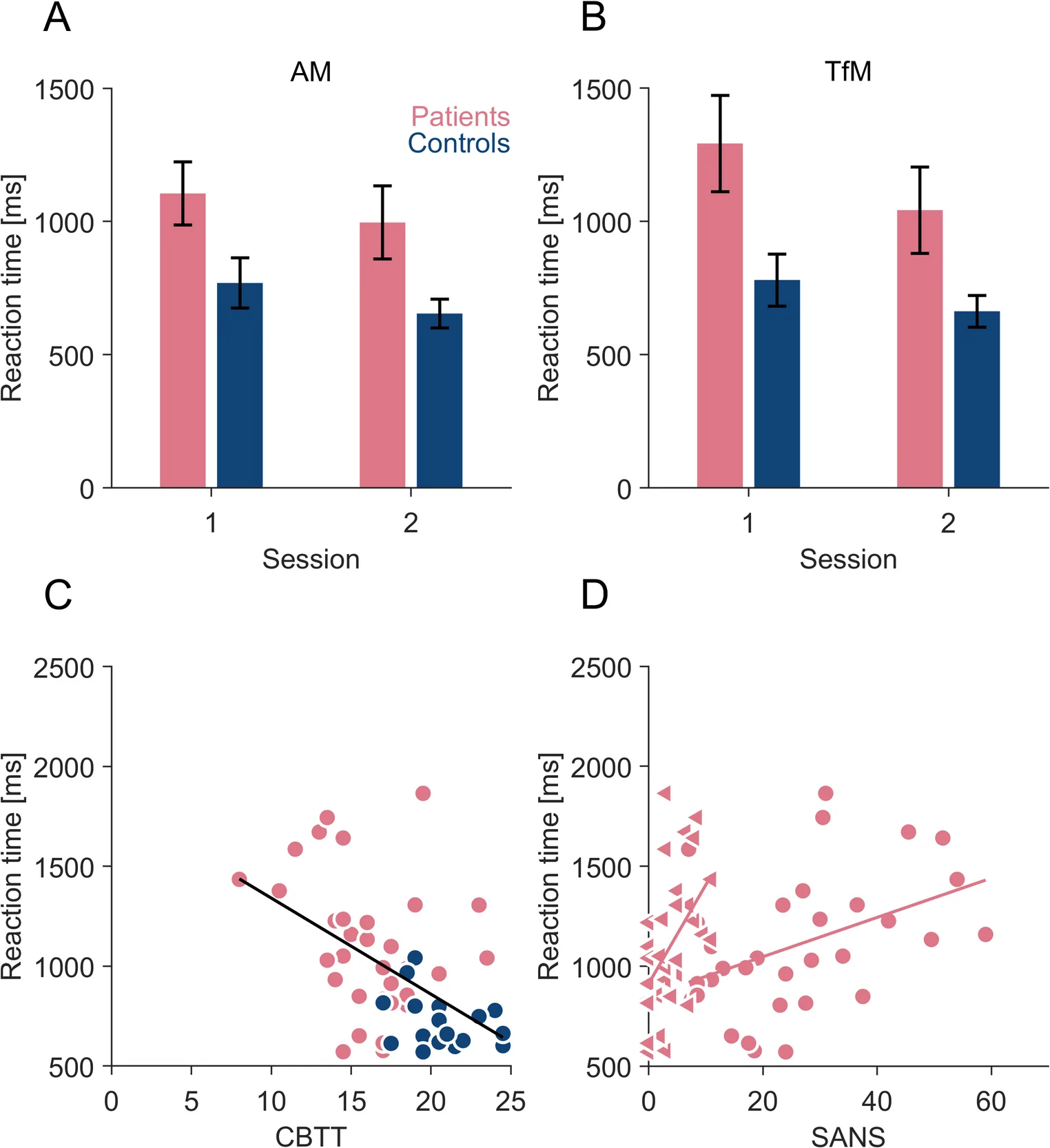
Reaction times. Red and blue indicate patients and controls, respectively. **A**, Apparent motion (AM) task. **B**, Transparency-from-motion (TfM) task. **C**, Relationship between reaction time and CBTT, averaged across tasks and sessions. **D**, Relationship between reaction time and SANS (circles) and SANS subscore IV (anhedonia – asociality) (triangles), respectively, all averaged across tasks and sessions. A & B, Error bars indicate 95% confidence intervals. C & D, Lines show linear fits.

Although the perceptual task was not speeded, we analysed reaction times as a measure of task difficulty. The average reaction times varied across all conditions between 654 ms ± 109.74 and 1292 ms ± 500.97 (Fig. 2). Using a Bayesian ANCOVA, the Bayes Factor (BF) indicated that the data best supported a model including effects of task, session, group and CBTT, with decisive evidence (BF_10_ = 2.65 × 10^16^) [42]. Post-hoc comparisons between AM (928.48 ms ± 325.37) and TfM (1006.73 ms ± 413.33) revealed posterior odds of 0.370 against the null model, indicating anecdotal evidence that reaction times were not substantially different between TfM and AM. Post-hoc comparisons between session 1 (1045.94 ms ± 375.27) and session 2 (889.27 ms ± 342.80) revealed posterior odds of 5.532 against the null model, indicating substantial evidence that reaction times were shorter in the second session. Post-hoc comparisons between patients (1108.86 ± 342.95) and controls (716.49 ± 131.50) revealed posterior odds of 2.05 × 10^9^ against the null model, indicating decisive evidence that patients responded slower than controls. Furthermore, there was a negative relationship between reaction time and CBTT averaged across tasks and sessions (r(48) = -0.52, *p* < 0.001), showing shorter reaction times for participants with better memory performance (Fig. 2C). For patients alone, the best model included effects of task, session and the SANS subscore IV (anhedonia – asociality) with decisive evidence (BF_10_ = 20206.71). There was a positive relationship between SANS subscore IV and reaction time averaged across tasks and sessions (r(30) = 0.49, *p* = 0.004), showing shorter reaction times for patients with lower SANS subscore IV (Fig. 2D). A similar relationship was present for the complete SANS (r(30) = 0.42, *p* = 0.016).

### Distribution of directional preferences

**Fig. 3:**
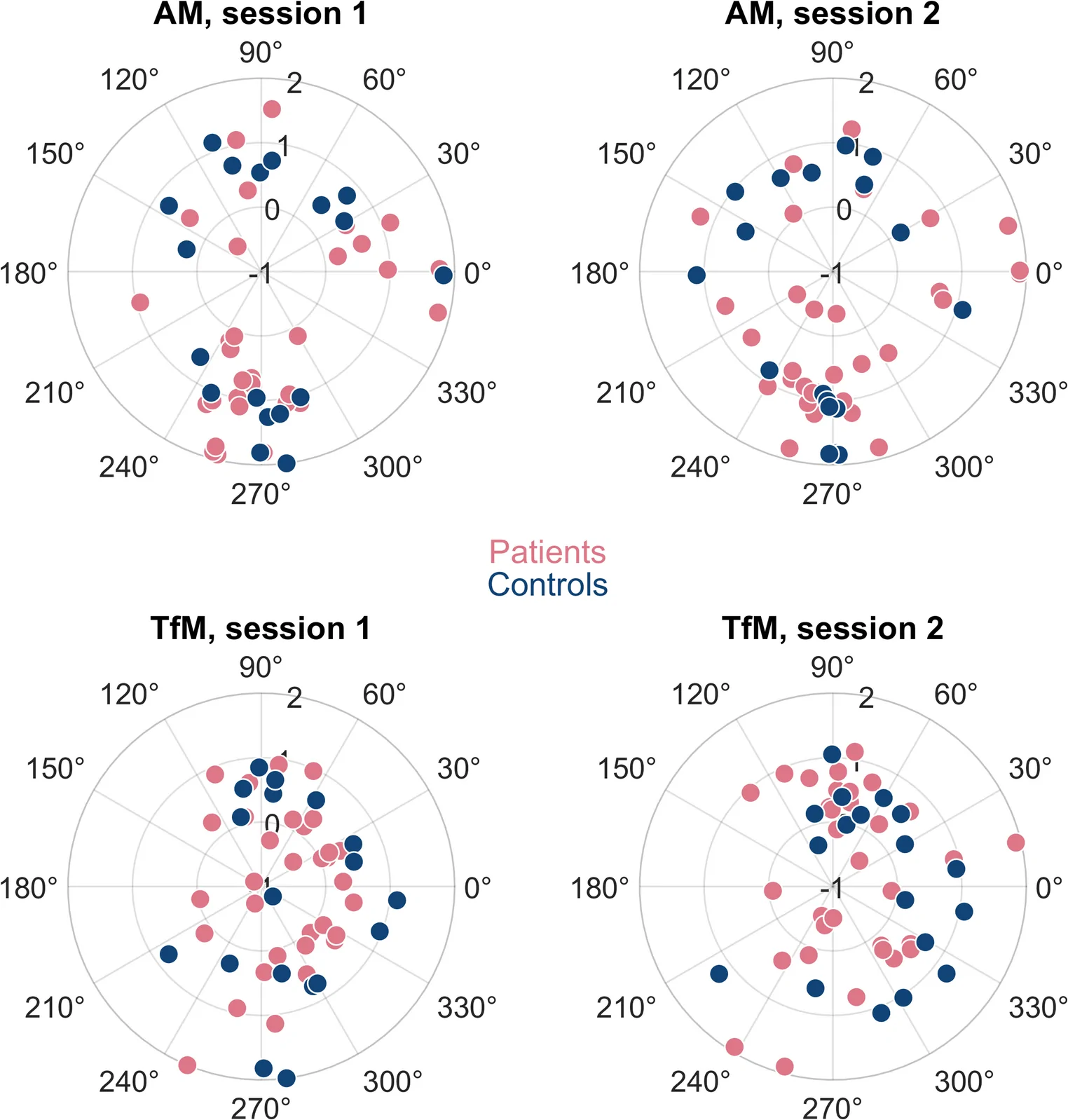
Distribution of directional preferences. The upper row shows data for apparent motion (AM) and the lower row for transparency-from-motion (TfM). The left column shows data from the first session and the right column from the second session. Each data point represents data from one participant. Red and blue indicate patients and controls, respectively. The polar angle and the radial location denote the preferred direction ($\:{\theta\:}_{m}$) and the preference strength (* b*) from Eq. 1, respectively. There was no correlation between the preferred directions and the preference strengths in the two tasks (Supplementary Information)

From the perceptual responses, we extracted a preference function with a preferred direction and a strength of that preference (Eq. 1, Fig. 1). While the preferred direction indicates which motion direction is perceived most frequently (AM) or most frequently in front (TfM), the preference strength indicates how strong this preference is and how sharp the transition between preferred and non-preferred directions is. The results show idiosyncratic directional preferences (Fig. 3), similar to previous reports with healthy participants [24, 23, 25, 22, 21].

### Strength of preferences

**Fig. 4:**
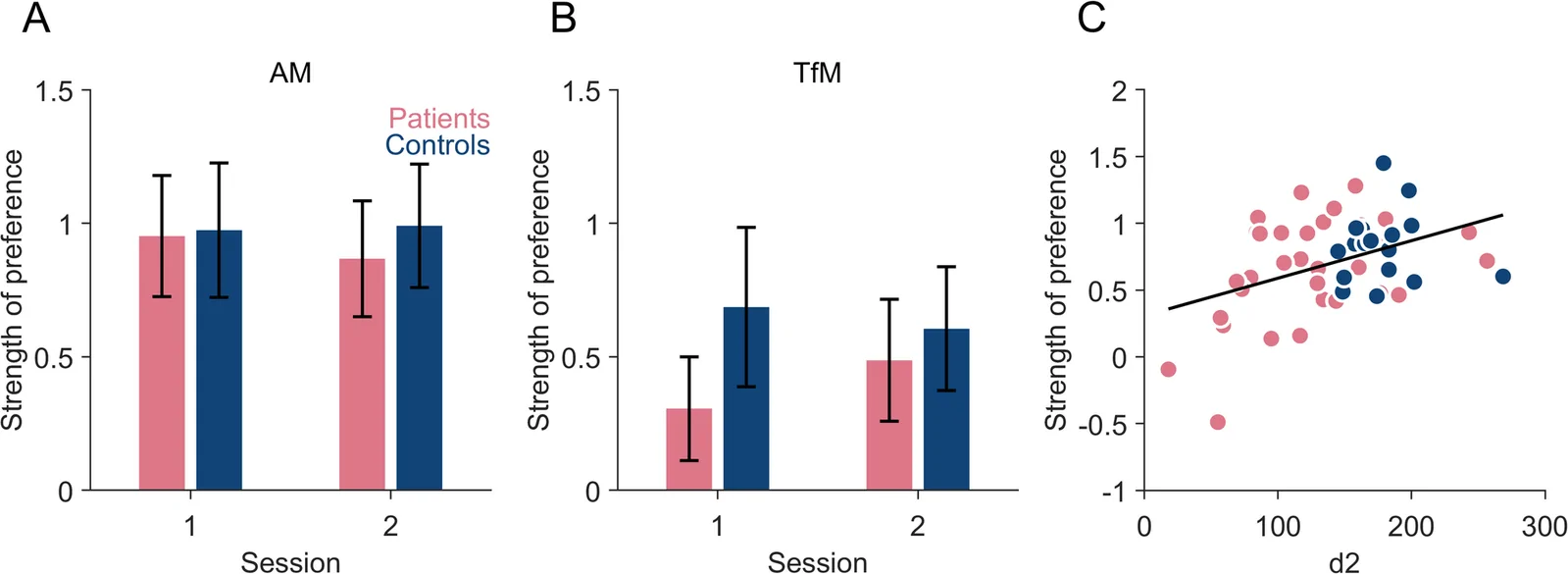
Preference strength. Red and blue indicate patients and controls, respectively. **A**, Apparent motion (AM) task. **B**, Transparency-from-motion (TfM) task. **C**, Relationship between the preference strength and d2 performance, averaged across tasks and sessions. The line shows a linear fit. A & B, Error bars indicate 95% confidence intervals.

The average preference strength (Eq. 1: parameter *b*; Fig. 4) varied across all conditions between 0.31 ± 0.54 and 0.99 ± 0.46. Using a Bayesian ANCOVA, the BF indicated that the data best supported a model including an effect of task and d2 with decisive evidence (BF_10_ = 3.86 × 10^7^). Post-hoc comparisons between AM and TfM revealed posterior odds of 129,628 against the null model, indicating decisive evidence that the strength was higher for AM that for TfM. The average strength was 0.95 ± 0.50 for AM and 0.49 ± 0.42 for TfM. Post-hoc comparisons between patients and controls revealed posterior odds of 0.70, indicating anecdotal evidence that there were no significant differences between patients and controls There was a positive relationship between d2 and the preference strength averaged across tasks and sessions (r(48) = 0.42, *p* = 0.003), indicating more pronounced preferences with higher d2 performance (Fig. 4C). For patients alone, the best model also included effects of experiment and d2 with decisive evidence (BF_10_ = 200190.38). Overall, preference strength was comparable in patients and healthy controls, but weaker preferences were related to attention deficits.

### Stability of preferences

To assess the temporal stability of the preference strength, we correlated the strength in session 1 and 2. For AM, there were significant correlations both for patients (r(30) = 0.57, *p* = 0.001) and healthy controls (r(16) = 0.62, *p* = 0.008). However, for TfM there was no significant correlation, neither for patients (r(30) = 0.32, *p* = 0.070), nor for healthy controls (r(16) = -0.24, *p* = 0.335). To analyse the stability of the preferred directions over time, we calculated circular correlation coefficients between the preferred directions in the two sessions. For patients, there was a significant correlation in both, AM (r(30) = 0.80, *p* = 0.002) and TfM (r(30) = 0.59, *p* = 0.002). For controls, there was only a significant correlation in TfM (r(16) = 0.60, *p* = 0.016), but not in AM (r(16) = 0.03, *p* = 0.876). In general, these correlations replicate previous findings [24, 21, 25] and indicate that there are idiosyncratic and stable directional preferences also in patients with SSD.

**Fig. 5:**
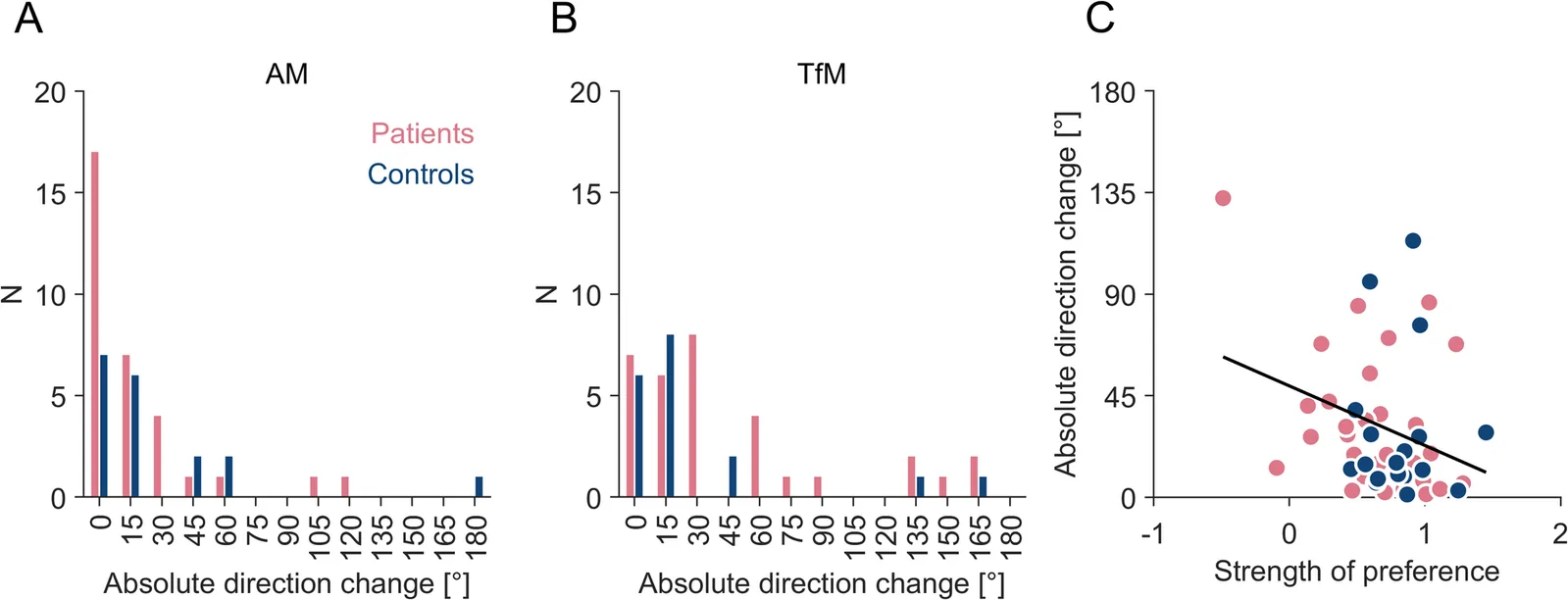
Stability of preferred directions. Red and blue indicate patients and controls, respectively. **A**, Apparent motion (AM) task. **B**, Transparency-from-motion (TfM) task. **C**, Relationship between the preference strength in the first session and the absolute change in preferred direction between the two sessions, averaged across tasks. The line shows a linear fit

Another way to assess the stability of preferences, is to calculate the absolute angular difference of the preferred direction in the two session (Fig. 5). If the preferences in the two sessions were completely independent of each other, one would expect an average difference of 90° (because the absolute differences can range between 0 and 180°). In AM, the change was on average 17.81° ± 27.96° and 27.29° ± 42.18° for the patients and controls, respectively. In TfM, the change was on average 45.13° ± 51.18 and 30.56° ± 45.67° for the patients and controls, respectively. All of these values were significantly smaller than 90° (all ps < 0.001). Using an ANCOVA (with interval between sessions, preference strength, d2 and CMBTT performance in the first session as covariates), the BF indicated that the data best supported a model including only an effect of preference strength in the first session with substantial evidence (BF_10_ = 3.944). There was a negative relationship between the preference strength in the first session and the absolute change of preferred direction across tasks (r(48) = -0.30, *p* = 0.033; Fig. 5C), indicating larger changes for weaker preferences. Post-hoc comparisons between patients and controls revealed posterior odds of 0.23, indicating substantial evidence that there were no significant differences between patients and controls. Overall, directional preferences were individually stable over time but fluctuations were larger for weaker directional preferences. Interestingly, the fluctuations were no more pronounced in patients than in controls, although the patients’ symptoms changed significantly over time.

## Discussion

We measured perceptual preferences in two ambiguous motion displays in patients with SSD and healthy participants. To assess the temporal stability of the directional preferences and their relationship to acute clinical symptoms, the preferences were measured twice, first during an acute phase and subsequently after remission. As expected, patients were more impaired in the first than second session, reflected by stronger positive and negative symptoms, lower performance in attention and memory tests and longer reaction times in the perceptual tasks. Sustained idiosyncratic preferences in the perceptual tasks were observed not only in healthy participants, but also in patients with SSD in both sessions. The preference strength was lower in TfM than in AM. In both cases, the preference strength was related to the performance in the d2-test of sustained attention. There were no other relationships with the magnitude of clinical symptoms or memory performance.

Our main finding is that patients with SSD had similar directional preferences as healthy controls, regarding the strength of preferences as well as their stability over time. While there are no consistent differences between patients and controls in the perception of geometric-optical illusions across studies [e.g. 43, 44, for a review see 15], patients have been shown to be less susceptible to depth-inversion illusions [e.g. 19, 18, for a review see 15]. Since the TfM stimulus includes an ambiguous depth order and therefore is related to the stimuli in depth-inversion illusions, one could have expected weaker preferences in patients than in controls in particular for this stimulus. These heterogeneous results indicate that perceptual anomalies in patients with schizophrenia might not be easily generalizable and instead might depend on the type of sensory stimulation and prior information. A potentially relevant factor might be the level of processing, with differences between low-level and high-level priors [12, 45]. The directional preferences for ambiguous depth order in TfM likely originate very early in visual processing based on a 1D motion signal [23]. Hence, these preferences might be located an even lower level than other low-level priors, such as the convexity prior or choice-history priors [45]. This is consistent with findings about serial dependency, which is intact in patients for stimuli with high sensory uncertainty [46, 47], but inverted for clear stimuli held in VWM [48]. Furthermore, patients are less able to update initial perceptual estimates with new information [49]. A recent study reported less stable precepts in an ambiguous structure-from-motion display in patients and unaffected siblings [50]. Since attentional performance was not assessed, it remains unclear if these differences relate to limitations in attention as in our results. The preservation of directional biases argues against a generalized alteration of priors in predictive coding frameworks, favouring rather selective alterations of high-level priors. The latter have been implicated in the formation of delusions and hallucinations.

Could methodological aspects have contributed to the stability of preferences? First, the regular medication of patients could have stabilized perceptual preferences [51, 52]. However, all clinical and performance measures showed clear differences between the acute and remitted session and it is unlikely that medication stabilized directional preferences exclusively. Furthermore, motion sensitivity was unaffected by medication in a previous study [51]. Second, the slightly shorter interval between the sessions in patients could have favored their stability. However, the interval had no significant influence on the changes in preferences between the two sessions, indicating that it is unlikely that it concealed actual differences in stability between patients and controls. Furthermore, preferences were equally stable over intervals of one, three or eight weeks in a previous study [21]. Finally, the statistical power of group comparisons is limited by the smaller size of the control group. However, reliable group differences in attention, VWM and reaction time, but not in directional preferences suggest sufficient power to detect typical differences between patients and controls. Furthermore, the Bayesian analysis provided substantial evidence that there were no differences between patients and controls in the stability of directional preferences.

The results indicated a close relationship between directional preferences and attentional resources. Across motion stimuli and groups, the preference strength and the performance in the d2-test were positively related. The d2-test measures the accuracy and speed of sustained visual scanning [53]. Directional preferences and sustained visual attention could be related in different ways: First, attention might limit the encoding/decoding of the directional preference. This would be conceptually similar to the effects of attention on performance in motion discrimination tasks of unambiguous, but noisy stimuli [54, 55] and the response gain of direction-selective neurons [56, 57]. Measuring the preference strength then corresponds to measuring thresholds in motion detection or discrimination tasks. In this case, a lack of attention should lead to shallower transitions between the preferred and the anti-preferred direction. Second, a lack of attention might lead to overall heightened distractibility, leading to a larger proportion of random responses overall. In this case, a lack of attention should not only result in a shallower transition between the preferred and anti-preferred direction but also to less biased responses at the preferred and the anti-preferred direction. Consistent with that perspective, attenuated visual center-surround interactions in patients have been attributed to lapses of attention [58]. Finally, higher distractibility could be related to higher volatility of directional preferences within a session, which would also result in a weaker preference overall. With the current data it is difficult to distinguish between these alternatives and future studies could employ an experimental manipulation of attention to establish a causal relationship. A previous study found correlated preference strength across multiple ambiguous motion displays, despite uncorrelated preferred directions [22]. Attention could be the limiting factor that drives these correlations. Further evidence for a crucial role of attention comes from the finding that the usefulness for a perceptual task can shift the preferred direction [59]. Our results indicate that attention can also affect the strength and/or the stability of the preference, which points to attention as a potential target for interventions aiming to stabilize perceptual processing.

Contrary to attention, VWM performance, as measured by the CBTT [36], was not related to the strength of the directional preferences. Perception of ambiguous stimuli has been related to memory on multiple time scales [60–62] and different types of memory might be involved: On the one hand, perception of ambiguous motion can be biased by contents of VWM [63]. On the other hand, perception depends on a retinotopic, perceptual memory [64, 65]. The absence of a relationship between memory performance and preference strength in our results could be interpreted in two ways: First, the limitation of memory in patients might not have been severe enough to affect the perception of the ambiguous stimuli. Second, the CBTT might rely on a different type of memory than the perception of ambiguous motion stimuli. VWM might be required in the ambiguous motion task to maintain the decision between the stimulus presentation and the manual response, which presumably is not challenging even for patients with memory deficits. The high level of performance with unambiguous control stimuli shows that patients had no problems to perform the task in general. Instead of VWM, some form of long-term perceptual memory might be involved in the directional preferences, since they were highly stable over an interval of about two months. However, VWM was negatively related to reaction time in the perceptual tasks. Reaction time was also positively related to the severity of negative symptoms. This indicates that the perceptual task was more challenging for patients with limitations of VWM and strong negative symptoms. Since the preference strength was related to performance in the d2-test rather than performance in the CBTT or negative symptoms, it seems to reflect a specific role of attention instead of a more general impairment.

While the main results were consistent for the two ambiguous motion displays, preferences were more rigid in AM than in TfM, as indicated by lower reaction times, and stronger and more stable directional preferences. This could originate from different causes of directional preferences in AM and TfM. The perceived direction in AM can be decoded from neuronal activity in area LIP [39] and from the direction of microsaccades before stimulus onset [66]. The preferences in depth ordering in TfM are determined by 1D motion signals [23] that arise before the aperture problem [67] is solved and that have been recorded in V1 [68] and early in area MT [69]. Although directional preferences for AM and TfM might originate from different neural substrates, they did not yield any differences with respect to schizophrenia.

To summarize, directional preferences in two ambiguous motion displays were comparable in healthy controls and patients with SSD. This demonstrates that patients in acute and remitted conditions, have intact long-term perceptual memory and can rely on perceptual priors to resolve sensory ambiguity similarly as healthy controls. Instead, the strength of directional preferences was limited by attentional resources, indicating that the reliable and internally consistent resolution of ambiguous motion signals requires attention.

## Supplementary Information

Below is the link to the electronic supplementary material.

Supplementary Material 1

## Data Availability

Anonymized data of the perceptual experiments and analysis scripts are available at https://doi.org/10.5281/zenodo.17276367.
