# Supplementary material for: The role of attention in perceptual biases in patients with schizophrenia spectrum disorder and healthy controls
Source: Eur Arch Psychiatry Clin Neurosci. 2026 Jul 4;276(7):3593–603. doi: 10.1007/s00406-026-02317-8 (PMC13562217; doi:10.1007/s00406-026-02317-8)
Supplement: Supplementary file 1 [file 406_2026_2317_MOESM1_ESM.pdf]

Supplementary Information: The role of attention in perceptual biases in patients with schizophrenia spectrum disorder and healthy controls

Alexander C. Schütz[1,2], Ogün Bolat[3], Kin Kori[3], Tilo Kircher[2,3], Benjamin Straube[2,3]

[1] AG Sensomotorisches Lernen, Fachbereich Psychologie, Philipps-Universität Marburg, Marburg, Germany
[2] Center for Mind, Brain and Behaviour, Philipps-Universität Marburg, Marburg, Germany
[3] Klinik für Psychiatrie und Psychotherapie, Philipps-Universität Marburg, Marburg, Germany

Corresponding author: Alexander C. Schütz, Philipps-Universität Marburg, Gutenbergstr. 18, 35037 Marburg

Keywords: Multistable perception; Schizophrenia; Priors; Individual differences; Motion perception.

Correlation between tasks

A fundamental question in perception of ambiguous displays and perceptual illusions is whether different effects can be traced back to a common factor [1–5]. To analyse the relationship between the two tasks, we correlated the reaction times, preferred directions and preference strength between AM and TfM. Since we did not expect any differences between patients and controls about how the two tasks are related to each other, we ran the analysis across the whole sample. The reaction times were highly correlated between the two tasks (session 1: $r(48) = 0.77$; $p < 0.001$; session 2: $r(48) = 0.68$; $p < 0.001$). There was no significant correlation between the preferred directions between AM and TfM (session 1: $r(48) = -0.22$; $p = 0.108$; session 2: $r(48) = -0.12$; $p = 0.384$). This replicates previous studies reporting that the preferred directions are not related in different types of ambiguous motion displays [2, 1]. Similarly, the preference strength were not significantly correlated (session 1: $r(48) = 0.13$; $p = 0.377$; session 2: $r(48) = 0.13$; $p = 0.380$). This is different from a large-scale online study, which reported significant correlations in the strength of directional preferences in AM and TfM [2].

References

1. Schütz AC (2014) Interindividual differences in preferred directions of perceptual and motor decisions. J Vision 14(12):16. https://doi.org/10.1167/14.12.16
2. Wexler M, Mamassian P, Schütz AC (2022) Structure of visual biases revealed by individual differences. Vision Res 195:108014. https://doi.org/10.1016/j.visres.2022.108014
3. Hamburger K, Hansen T (2010) Analysis of individual variations in the classical horizontal-vertical illusion. Attention, perception & psychophysics 72(4):1045–1052. https://doi.org/10.3758/APP.72.4.1045
4. Cretenoud AF, Karimpur H, Grzeczkowski L, Francis G, Hamburger K, Herzog MH (2019) Factors underlying visual illusions are illusion-specific but not feature-specific. J Vision 19(14):12. https://doi.org/10.1167/19.14.12
5. Grzeczkowski L, Clarke AM, Francis G, Mast FW, Herzog MH (2017) About individual differences in vision. Vision Res 141:282–292. https://doi.org/10.1016/j.visres.2016.10.006
